# Histone H2A C-Terminus Regulates Chromatin Dynamics, Remodeling, and Histone H1 Binding

**DOI:** 10.1371/journal.pgen.1001234

**Published:** 2010-12-09

**Authors:** Christine Vogler, Claudia Huber, Tanja Waldmann, Ramona Ettig, Lora Braun, Annalisa Izzo, Sylvain Daujat, Isabelle Chassignet, Andres Joaquin Lopez-Contreras, Oscar Fernandez-Capetillo, Miroslav Dundr, Karsten Rippe, Gernot Längst, Robert Schneider

**Affiliations:** 1 Max-Planck Institute of Immunobiology, Freiburg, Germany; 2 Biochemie III, University of Regensburg, Regensburg, Germany; 3 German Cancer Research Center (DKFZ) and BioQuant, Research Group Genome Organization and Function, Heidelberg, Germany; 4 Genomic Instability Group, Spanish National Cancer Research Centre, Madrid, Spain; 5 Rosalind Franklin University, North Chicago, Illinois, United States of America; University of Cambridge, United Kingdom

## Abstract

The tails of histone proteins are central players for all chromatin-mediated processes. Whereas the N-terminal histone tails have been studied extensively, little is known about the function of the H2A C-terminus. Here, we show that the H2A C-terminal tail plays a pivotal role in regulating chromatin structure and dynamics. We find that cells expressing C-terminally truncated H2A show increased stress sensitivity. Moreover, both the complete and the partial deletion of the tail result in increased histone exchange kinetics and nucleosome mobility *in vivo* and *in vitro*. Importantly, our experiments reveal that the H2A C-terminus is required for efficient nucleosome translocation by ISWI-type chromatin remodelers and acts as a novel recognition module for linker histone H1. Thus, we suggest that the H2A C-terminal tail has a bipartite function: stabilisation of the nucleosomal core particle, as well as mediation of the protein interactions that control chromatin dynamics and conformation.

## Author Summary

Histones are the main protein components of chromatin. The N-terminal tails of histones stick out from the nucleosomes, the building blocks of chromatin, and are involved in the regulation of all DNA–dependent processes. Only Histone H2A has an additional C-terminal tail and currently very little is known about the function of this tail. The H2A C-terminus protrudes from the nucleosome and is located where the DNA enters and leaves the nucleosome. We show here that it can interact with the linker histone H1 that is important for higher order chromatin structure. We also find that this tail is involved in regulating nucleosome dynamics and mobility of H2A itself. The C-terminal H2A tail has also an important function in regulating the activity of chromatin remodelers, enzymes that can reposition nucleosomes. Furthermore we find that cells expressing C-terminally truncated H2A are more sensitive to stress, demonstrating that this tail is important for cellular homeostasis. Together our results reflect a key function of the H2A C-terminus in chromatin biology.

## Introduction

In the eukaryotic nucleus, DNA is stored in a nucleoprotein complex referred to as chromatin. This packaging of DNA does not only serve to condense DNA into a highly compacted form, it is also fundamental for the regulation of all DNA-dependent processes such as transcription, replication and DNA repair [1]. The basic unit of chromatin is the nucleosome where 147 bp of DNA are wrapped around an octamer of two copies of the four core histones H2A, H2B, H3 and H4 [2]. The flexible N-terminal tails of the core histones can be extensively post-translationally modified, and have been shown to play important roles in chromatin structure and transcriptional regulation [3].

Accessibility to DNA sequences occluded by nucleosomes can be regulated by chromatin remodeling complexes. These enzymes use energy from ATP hydrolysis to disrupt contacts between the histone octamer and the DNA and thus move nucleosomes along the DNA. Many of these complexes are conserved from yeast to human. Chromatin remodeling factors can be divided into four main classes according to their ATPase subunit: Swi2, ISWI, Mi-2/CHD and INO80 [4]. By modulating chromatin fluidity they are not only necessary for transcription, but for all DNA-dependent processes such as replication, recombination and DNA repair [5], [6].

In the core nucleosome, the H3 – H4 tetramer is located in the centre with the two H2A – H2B dimers being positioned at the nucleosomal DNA entry-exit sites [2]. In this complex H2A and H2B dissociate more easily from the nucleosome than H3 and H4 [7], [8]. The fifth histone, linker histone H1, can associate with linker DNA as well as with the nucleosome, via its globular domain and its C-terminus leading to the formation of chromatosomes and a tighter packaging of chromatin [9]. However, the precise binding site of H1 is not known, although several models exist for the interaction of the globular domain and full length linker histone with the nucleosome core particle at the DNA entry and exit [10]–[15].

H2A is the only core histone that contains an additional flexible C-terminal extension besides the N-terminal tail. Whereas the N-terminal histone tails have been investigated in numerous studies, very little is known about the function of the H2A C-terminal tail. The tail consists of 15 amino acids beyond the globular domain, it is unstructured and leaves the nucleosome at the entry-exit site of the nucleosomal DNA [2]. It can be divided into two parts: aa 115–122 pass between the strands of DNA wrapped around the nucleosome; aa 123–129 protrude from the nucleosomal structure [2]. In isolated nucleosomes the end of the tail contacts DNA at the dyad axis of the nucleosome but it is rearranged towards the edge of nucleosomal DNA when linker DNA is present, suggesting a DNA-dependent rearrangement [16].

In this study, we have addressed for the first time the biological function of the canonical H2A C-terminal tail. We show that cells expressing C-terminally truncated H2A were sensitive to certain types of cellular stress, suggesting an important role of this tail in cellular homeostasis. In line with this we demonstrate a key role for the H2A-C terminal tail in nucleosome stability and mobility *in vivo* and *in vitro*. Furthermore, we show a functional role of this C-terminal H2A tail in regulating chromatin remodeling by ISWI-type remodelers. Finally, we reveal a novel function of the C-terminal tail of H2A in modulating binding of linker histone H1 to the nucleosome.

## Results

### *In vivo* stability and mobility

**Figure 1:**
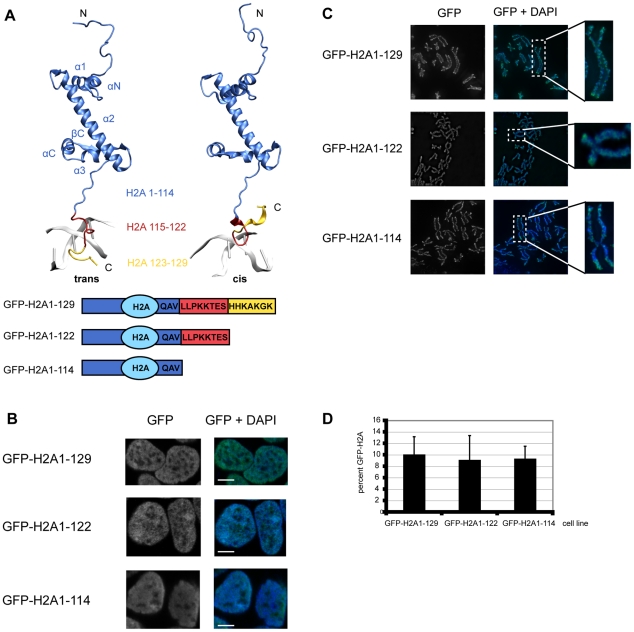
The C-terminal tail of canonical H2A. (A) Structure of histone H2A. Top: Histone H2A extracted from the crystal structure of the nucleosome core particle (1kx5) [58]. Residues of H2A are depicted according to the following color code: H2A1-114 blue, H2A115-122 red, H2A123-129 yellow. The C-terminal tail can be present in a *trans* or *cis* conformation with respect to the βC part of the H2A histone. Transitions between the two conformations occur on the 100 ns time scale as inferred from MD simulations (Ettig & Rippe, data not shown). Bottom: Schematic respresentation of the H2A constructs used. (B) GFP-H2A fusion proteins in HEK293 cells stably expressing GFP-H2A1-129, GFP-H2A1-122 or GFP-H2A1-114 localize to the nucleus. Immunoflourescence images of representative cells are shown. The DNA was counterstained with DAPI. The scale bar represents 5 µm. (C) GFP-H2A fusions are present on metaphase chromosomes of HEK293. Metaphase chromosomes were prepared from cells stably expressing GFP-H2A1-129, GFP-H2A1-122 or GFP-H2A1-114. Representative examples are shown. The DNA was counterstained with DAPI. (D) Expression level of GFP-H2A fusion proteins is 5–10% of endogenous H2A. Quantitative Western blot analysis of the GFP-H2A signal compared to the endogenous H2A signal using an antibody against H2A. The percentage of GFP-H2A is shown, error bars represent s.d.

The H2A C-terminal tail protrudes from the nucleosomal core particle, is unstructured and comprises the 15 amino acids (aa) C-terminal to the H2A globular domain. In our molecular dynamics simulations (MD) of histone-DNA interactions we find that the H2A C-terminus adopts two types of conformation (*cis* and *trans*) that are distinct with respect to their interactions with the linker DNA (Figure 1A). In the *trans* conformation 5–6 amino acids of the H2A C-terminus contact on average a region of 10 DNA nucleotides in the major groove, as opposed to interactions between 3–4 amino acids with 8 minor groove DNA residues in the *cis* conformation. Transitions between the two conformations occur on a typical time scale of 50–100 ns (data not shown).

To elucidate the biological function of the H2A-C terminal tail *in vivo*, we generated HEK293 cell lines stably expressing N-terminal GFP fusions of H2A1-129 (full-length) and the two C-terminally truncated proteins H2A1-122 (lacking the protruding region) and H2A1-114 (lacking the complete tail, including the ubiquitination site at K119) (Figure 1A). Histone-GFP fusions have been previously employed successfully to study chromatin by numerous researchers [17]–[19]. Indeed, GFP fusions of histones can genetically complement for the loss of the endogenous gene in *Tetrahymena* [20] and a ChIP-Seq study did not detect any significant differences in the genome-wide localization of H3.3-HA and that of H3.3-EYFP [18]. Recent data did not reveal functional differences between N-terminally and C-terminally GFP-tagged H2A [21] and showed that GFP-H2A fusions are incorporated into nucleosomes *in vivo* [22]. As expected, our GFP-H2A proteins were exclusively present in the nucleus (Figure 1B), found in mitotic chromosomes (Figure 1C) and were incorporated normally into chromatin (Figure S1A). The GFP-signals were scattered throughout the nucleoplasm (Figure 1B) following the DNA density with no preferential localisation to euchromatin or heterochromatin (Figure S1B). The protein levels of GFP-H2A were about 10% of the endogenous H2A protein in all three cell lines (Figure 1D), an expression level of ‘exogenous’ histones that has been shown to be non-deleterious and not to cause unspecific defects [23].

### The effect of C-terminal truncations on cells

**Figure 2:**
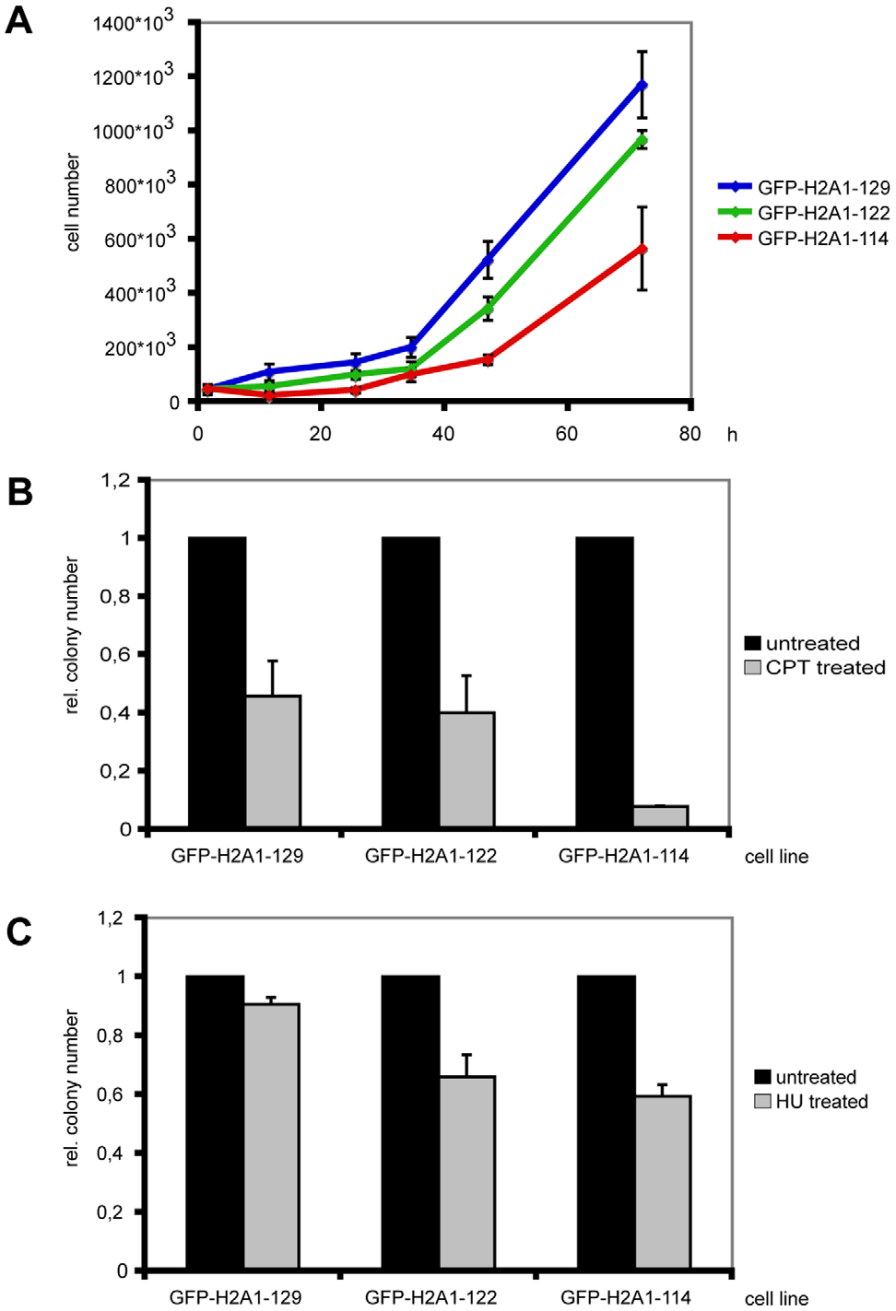
Cells expressing C-terminally truncated GFP-H2A are stress sensitive. (A) Synchronized cells expressing GFP-H2A1-122 and GFP-H2A1-114 show a reduced growth rate. Cells were seeded at a density of 5*10^3^ cells ml^−1^ and subjected to a double thymidine block. Cell numbers were determined in triplicates approx. every 24 h for 3 d after release from the block. Growth curves of one representative experiment are shown with standard deviations. (B) Cells expressing GFP-H2A1-114 are more sensitive to CPT treatment. After treatment with 1 µM CPT for 30 min, a colony forming assay was performed and colonies were fixed and stained with crystal violet. The colony number was determined and the colony number for untreated cells was set to 1. The experiment was performed in triplicates and average relative colony numbers with standard deviation of one representative experiment are given. (C) Cells expressing GFP-H2A1-122 and GFP-H2A1-114 are more sensitive to HU treatment. Cells were treated with 1 mM HU for approx. 4 h. Colony numbers were determined as in 2B.

Since alterations in histone composition have been shown to have an impact on cellular proliferation [23] we asked how expression of the GFP-H2A truncations affects these processes. To obtain a homogenous cell population we first synchronized the cells by a double thymidine or nocodazole treatment and then measured growth curves after release. We observed a significant reduction in growth rate for C-terminally truncated H2A as compared to cells expressing full-length GFP-H2A (Figure 2A and data not shown).

Since the thymidine block can cause accumulation of DNA damage [24] we investigated if cells expressing C-terminally truncated H2A are indeed sensitive to DNA damage. For this we treated these cells with the DNA damage-inducing reagent camptothecin (CPT), a topoisomerase I inhibitor that introduces DNA strand breaks by preventing DNA religation. After CPT treatment cells expressing H2A1-114 showed an altered sensitivity towards CPT as reflected by a strong decrease in colony forming ability (Figure 2B). This result is in line with an observed reduction in colony forming ability after the double thymidine treatment (data not shown).

To further investigate the sensitivity of cells expressing C-terminally truncated H2A to stress, we treated cells with the ribonucleotide reductase inhibitor hydroxyurea (HU). Cells expressing C-terminally truncated H2A showed altered sensitivity towards this damaging agent and the number of colonies formed was reduced as compared to cells expressing full-length GFP-H2A (Figure 2C). The cells expressing truncated H2A were also more sensitive to exposure to non-genotoxic stress such as osmotic stress compared to cells expressing full-length GFP-H2A (data not shown).

### The role of the H2A C-terminus in nucleosome stability *in vivo*

**Figure 3:**
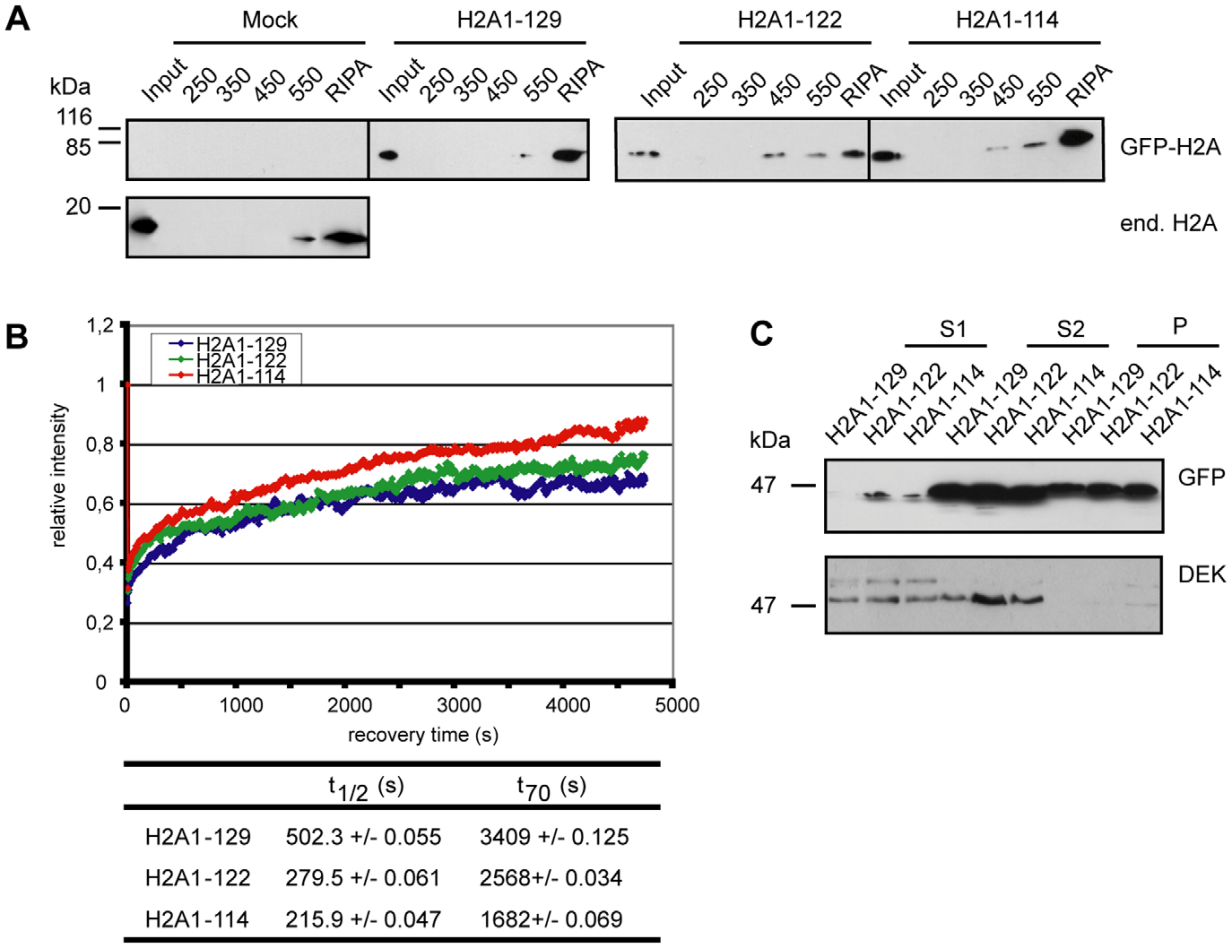
Loss of the H2A C-terminal tail negatively affects nucleosome stability. (A) Nucleosomes containing C-terminally truncated H2A elute at lower salt concentrations. Stepwise salt elution of GFP-H2A fusion proteins from HEK293 cells stably expressing GFP-H2A1-129, GFP-H2A1-122 or GFP-H2A1-114. Extracts from 2*10^5^ cells per lane were analyzed by immunoblot with antibodies against endogenous H2A or GFP as indicated. (B) Deletion of the H2A C-terminus results in increased FRAP recovery kinetics. FRAP analysis with stable cell lines expressing GFP-H2A1-129, GFP-H2A1-122 and GFP-H2A1-114. Left panel: A 120×120 pixel spot was bleached and the fluorescence recovery was measured in 5.01 s intervals over 80 min. 10–15 cells were used for quantification. The recovery curve for GFP-H2A1-129 is in blue, for GFP-H2A1-122 in green and for GFP-H2A1-114 in red. Bottom panel: Calculated t_1/2_ and t_70_ recovery times. (C) C-terminally truncated H2A is enriched in accessible chromatin fractions. For chromatin fractionation, approx. 1*10^8^ nuclei from stable cell lines expressing GFP-H2A1-129, GFP-H2A1-122 and GFP-H2A1-114 were digested with MNase for 1 min and fractionated into S1, S2 and P (pellet). The fractions were precipitated and 1/50 (S1 and S2) or 1/100 (P) was analyzed by immunoblotting with a GFP specific antibody (upper panel). As a control an immunoblot against the chromatin associated protein DEK was used (lower panel).

Since histone tails can regulate nucleosome mobility and stability [25] the altered stress sensitivity could be due to alterations in nucleosome dynamics. We thus next addressed whether the H2A C-terminal tail affects nucleosome stability *in vivo* by performing a stepwise salt elution of proteins from nuclei prepared from the cells expressing GFP-H2A1-129, GFP-H2A1-122 and GFP-H2A1-114 [17]. As histone proteins are bound very tightly to chromatin, only a small amount of endogenous H2A was found in the 550 mM NaCl fraction, and the bulk of protein could only be released using RIPA buffer (Figure 3A). As expected, we observed the same for full-length GFP-H2A. In contrast, GFP-H2A1-122 and GFP-H2A1-114 were present already in the 450 mM salt fraction. Notably, in this fraction we did not detect endogenous H2A or full-length GFP-H2A. This indicates that a fraction of nucleosomes containing C-terminally truncated H2A is less stable than wild-type H2A-containing nucleosomes.

To find out whether the loss of nucleosome stability due to the H2A C-terminal truncation results in increased histone mobility, we performed fluorescence recovery after photobleaching (FRAP) experiments (Figure 3B) [26]. Core histones display a relatively low mobility with recovery rates on the minutes to hour scale [27] as reflected here in the observed t_70_ recovery time of 57 min for full-length GFP-H2A (Figure 3B bottom panel). In contrast, deletion of the complete C-terminal tail drastically increased the mobility of GFP-H2A to a t_70_ recovery time of only 28 min, i.e. ∼50% of the full-length counterpart, comparable to the results of Higashi et al. [21], which used a larger deletion. The partial truncation GFP-H2A1-122 showed an intermediate mobility with a t_70_ recovery rate of 43 min (Figure 3B right panel), suggesting that also the part of the tail that protrudes from the nucleosome is important for the dynamics of H2A chromatin integration. Together these experiments show that the H2A C-terminus plays a key role in determining nucleosome stability and H2A mobility *in vivo*.

We next aimed to investigate the global distribution and the localization of the truncated H2A proteins within functionally distinct chromatin domains. For this, we digested nuclei prepared from our stable cell lines with MNase I and fractionated the chromatin [28], [29]. We first collected the S1 supernatant fraction, which is enriched in transcriptionally active, accessible chromatin domains (Figure 3C). The remaining nuclear pellet was then further fractionated into the supernatant S2 enriched in untranscribed, compacted chromatin, and the pellet P containing insoluble chromatin [29]. Interestingly, we found both GFP-H2A1-122 and GFP-H2A1-114 in the S1 fraction after a short MNase I treatment (Figure 3C, upper panel) indicating that they can associate with accessible regions of chromatin. In contrast, we detected full-length GFP-H2A in the S1 fraction only after prolonged MNase I digestion. These results show that H2A without its C-terminal tail is enriched in the accessible chromatin fraction, consistent with our observations of higher mobility of nucleosomes containing truncated H2A.

### The role of the H2A C-terminus in nucleosome assembly and dynamics

**Figure 4:**
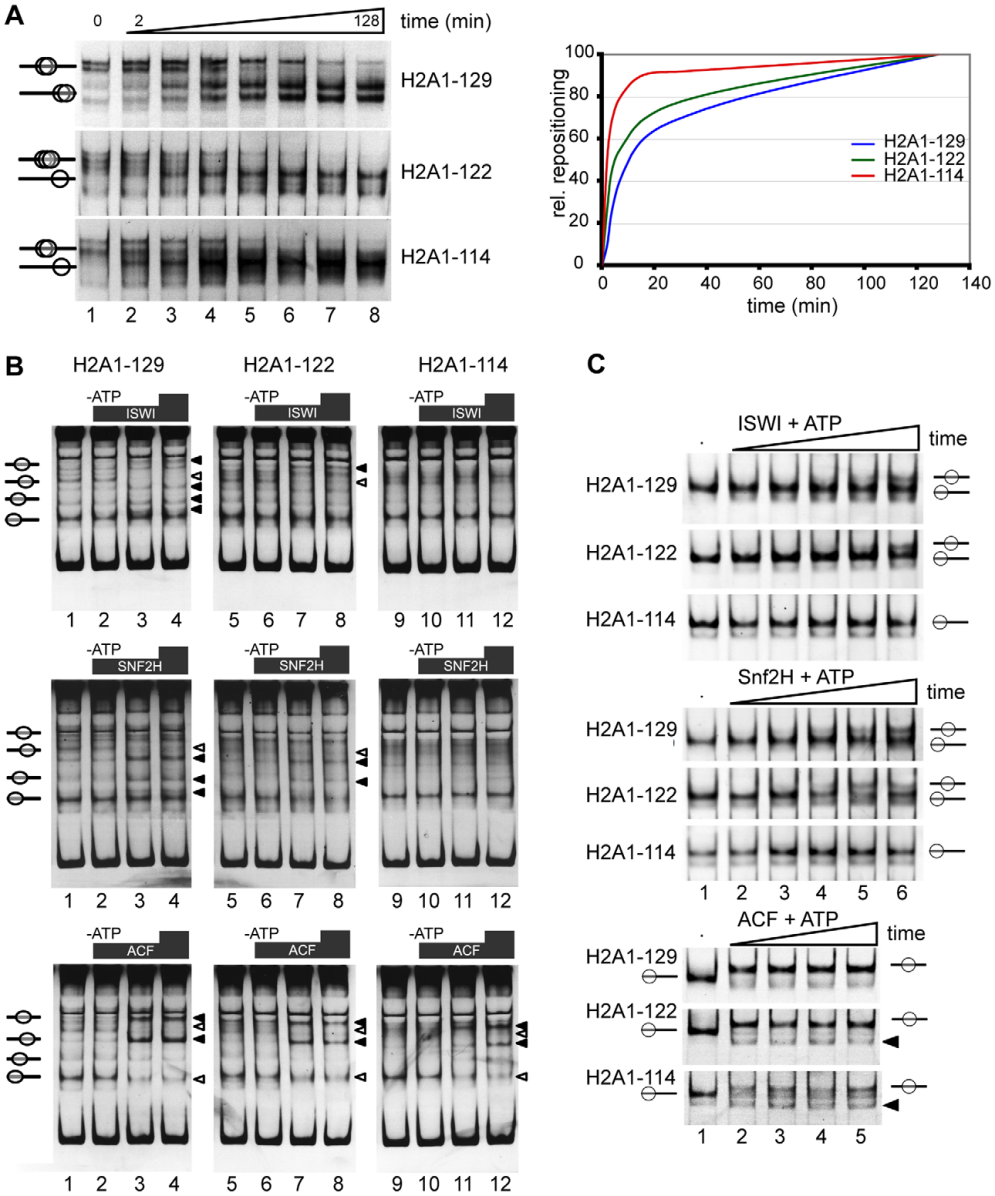
The H2A C-terminal tail is important for nucleosome stability and chromatin remodeling in vitro. (A) Differential positioning and thermal shift of mononucleosomes containing either full-length H2A, H2A1-122 or H2A1-114. Nucleosomes were reconstituted on a linear MMTV NucA DNA fragment and incubated at 45°C for increasing periods of time. Nucleosome positions were analyzed on a native 5% polyacrylamide gel and visualized by ethidium bromide staining (left panel). Quantification of the relative repositioning is shown in the right panel. (B) C-terminal deletions affect chromatin remodeling. *In vitro* chromatin remodeling assay with mononucleosomes containing H2A1-129, H2A1-122 or H2A1-114 that were assembled on linear DNA fragment (*Drosophila* Hsp70 promoter) and incubated with the indicated chromatin remodeling complex with or without ATP. Upper panel: ISWI. Middle panel: SNF2H. Lower panel: ACF. Lanes 1, 5, 9: reconstituted mononucleosomes alone; lanes 2, 6, 10: + remodeling factor, no ATP; lanes 3, 7, 11 and 4, 8, 12: + remodeling factor, + ATP. Nucleosome positions were analyzed by native gel electrophoresis and staining with ethidium bromide. Black arrowheads indicate positions to which nucleosomes were moved, white arrowheads indicate positions from which nucleosomes were removed. (C) Kinetics of nucleosome remodeling assayed with a 601 remodeling template. Nucleosomes containing the wildtype H2A and the indicated C-terminal truncations (300 ng), as indicated on the left, were incubated without (lane 1), or with 100 ng of the indicated remodeling enzyme and ATP (lanes 2 to 6). The remodeling reaction was incubated from 1 up to 40 min and analyzed as described above. Nucleosome positions are indicated. Black triangles indicate new nucleosome positions observed with H2A1-122 and H2A1-114.

To gain further mechanistic insights in the functions of the H2A C-terminus, we used an *in vitro* system with recombinant human histone octamers containing either full-length H2A1-129, H2A1-122 or H2A1-114. We analyzed the positioning and thermal mobility of these mononucleosomes reconstituted on a short linear DNA fragment with a central NucA positioning sequence from the MMTV long terminal repeat [30]. As depicted in Figure 4A the initial positioning on the DNA fragment was different for nucleosomes with wild-type H2A compared to those with C-terminal truncations of H2A. Differential positioning was also observed with other nucleosome positioning sequences (data not shown). Thus, the C-terminal tail contributes to the stabilization and the selection of specific nucleosome positioning sequences.

To determine whether the H2A tail influences thermal mobility of nucleosomes, we examined the kinetics of nucleosome repositioning after incubation at increased temperature (45°C) [30]–[32]. Deletion of the H2A C-terminal tail increased thermal mobility, with H2A1-114 containing nucleosomes moving considerably faster towards the terminal position than wild-type nucleosomes (Figure 4A). The smaller deletion in H2A1-122, where the DNA passing region of the tail is still present, resulted in a less pronounced increase of thermal mobility (Figure 4A). Together these data reveal a higher mobility of nucleosomes lacking the C-terminus of H2A and highlight a potential role for the H2A C-terminus in nucleosome positioning.

### Truncation of the H2A C-terminus affects chromatin remodeling

Given the important contribution of the H2A C-terminal tail to nucleosome mobility and positioning observed here, the question arises whether this tail also affects nucleosome positioning by ATP-dependent remodeling factors. Thus, we reconstituted mononucleosomes on two different linear DNA fragments: the *Drosophila* Hsp70 promoter sequence [33] yields a complex pattern of multiple nucleosome positioning sites, that allow us to monitor remodeling and positioning differences (Figure 4B), whereas the 601 remodeling template is well suited to follow the kinetics of the remodeling reaction (Figure 4C). We performed chromatin remodeling reactions with recombinant human SNF2H or *Drosophila* ISWI and ACF. All three remodelers belong to the ISWI-family of ATPases. SNF2H is the human orthologue of the *Drosophila* ISWI ATPase and ACF is a complex of the ISWI ATPase and the Acf1 subunit [34],[35]. On the Hsp70 substrate new nucleosomal positions (Figure 4B, black triangles) appeared while other nucleosomal bands disappeared (Figure 4B, white triangles). The remodeling behaviour was strongly dependent on the remodeling ATPase used, as specific remodeling machines have different positioning properties [36]. Surprisingly, the remodeling efficiency of ISWI and SNF2H was clearly reduced for nucleosomes containing H2A1-114 compared to full-length H2A or H2A1-122 (Figure 4B) suggesting that amino acids 114 to 122 are directly involved in the remodeling reaction. However, ACF, a complex with higher remodeling efficiency as compared to the isolated ATPase motor proteins [37] was able to reposition nucleosomes with C-terminally truncated H2A1-114. To investigate the differences in the remodeling kinetics we also analyzed the reaction with 601 DNA nucleosomes (Figure 4C). As observed with the Hsp70 substrate, ISWI and SNF2H were efficiently remodeling the H2A1-122 and wildtype H2A containing nucleosomes. Again, both remodelers were not capable to efficiently relocate H2A1-114 containing nucleosomes during the time course analyzed. In contrast ACF translocated nucleosomes with similar kinetics for all three substrates, albeit with differences in the final positions.

Thus, our data show that the H2A C-terminal tail is required for facilitating efficient ATP-dependent nucleosome repositioning by SNF2H and ISWI. Only the highly effective remodeling machine ACF, containing two motor subunits, can overcome this obstacle to the remodeling reaction [35]. In addition we observed that similar to the results of the thermal mobility assay, the H2A C-terminus is required for the establishment of defined nucleosome positions on the various substrates examined.

### The C-terminus of H2A interacts with histone H1

**Figure 5:**
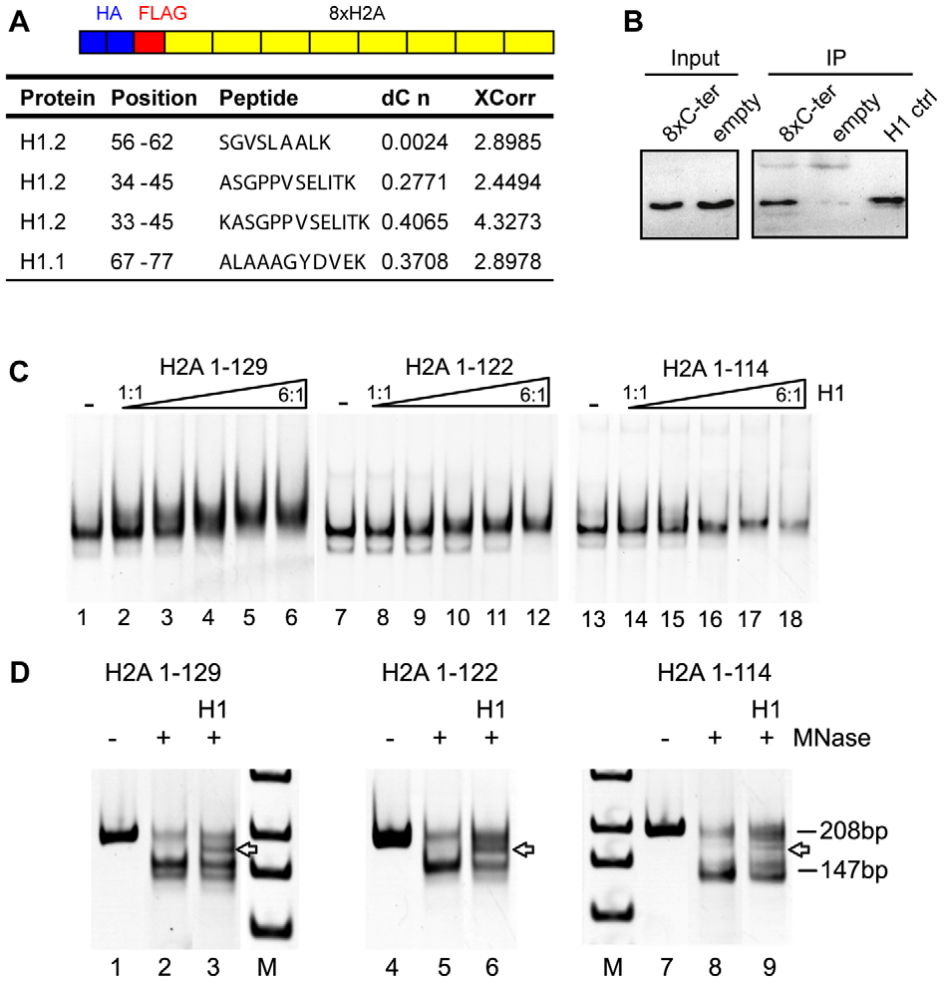
The H2A C-terminal tail binds linker histone H1. (A) Schematic representation of the construct used to identify H2A C-terminus interacting proteins. Proteins interacting with the HA Flag 8x C-terminus were identified by Mass Spectrometry after Flag affinity purification. Histone H1 peptides that bind to the H2A C-terminal tail as identified by mass spectrometry. DeltaCN and XCorr values are shown. (B) H1 co-immunoprecipitates with the 8x repeat of the H2A C-terminal tail. The HA-Flag 8x H2A C-terminus was purified and the eluate from the Flag Sepharose probed by immunoblotting with an H1 specific antibody. 10% of the input was loaded (lanes 1 and 2). As control, the empty vector without the 8x H2A C-terminus was used. Purified H1 was used as an immunoblot control (lane 5). (C) Deletion of the H2A C-terminus reduces H1 binding. Binding of H1 to *in vitro* reconstituted mononucleosomes containing either full-length or C-terminally truncated H2A. Mononucleosomes containing wild type H2A (lanes 1–6), H2A 1-122 (lanes 7–12) or H2A 1-114 (lanes 13–14) were reconstituted on a DNA fragment containing the 601 positioning sequence. The nucleosomes were incubated with increasing amounts of H1 (H1 to nucleosome ratios 1∶1 to 6∶1) and then analyzed on native polyacrylamide gels. Nucleoprotein complexes were visualized by ethidium bromide staining. (D) Partial MNase digestion of H1-nucleosome complexes shows a chromatosome stop. Nucleosomes and H1-nucleosome complexes reconstituted on a 208 bp 601 DNA fragment at a molar ratio of 6∶1 were incubated with increasing MNase concentrations and the resulting DNA cleavage products were analyzed by polyacrylamide gel electrophoresis and ethidium bromide staining next to a DNA standard (M). The position of the undigested DNA fragment (208 bp), the protected nucleosomal DNA (147 bp) and the chromatosome stop (arrow) are indicated.

Next, we explored the potential role of the H2A C-terminal tail as a recruitment platform for specific binding proteins since it protrudes from the nucleosome, which could facilitate protein interactions. To identify interaction partners we performed an unbiased screen. For this we generated HeLa cells stably expressing eight repeats of the H2A C-terminus and identified interacting proteins by mass spectrometry (Figure 5A). Several peptides were detected that corresponded to histone H1.1 and H1.2 (Figure 5A) but no peptides originating from core histones. We confirmed the interaction between histone H1 and the H2A C-terminal tail repeats by co-immunoprecipitation (Figure 5B). Next we wanted to know if deletion of the C-terminal H2A tail indeed affects H1 binding to the nucleosome. Thus, we conducted binding studies of H1 to nucleosomes reconstituted on a DNA fragment of 208 bp containing the 601 nucleosome positioning sequence [38]. Incubation of the nucleosomes containing wildtype H2A with increasing amounts of H1 resulted in a super-shift, starting to appear at equimolar H1 to nucleosome ratios (Figure 5C, lanes 1 to 6). Importantly, we found decreased binding of H1 to mononucleosomes lacking the H2A C-terminus as compared to wild type nucleosomes (Figure 5C, compare lane 3 with 9 and 15), suggesting that the H2A C-terminus is required for efficient H1 binding.

In order to test whether the weaker H1 interaction with nucleosomes lacking the H2A C-terminus can result in the formation of a bona fide chromatosome, we studied the H1 binding mode by nuclease digestion. Micrococcal nuclease digestion of the nucleosomes reconstituted with wildtype H2A and C-terminally deleted H2A resulted in the appearance of an intermediate size DNA fragment of about 160 bp (Figure 5D, arrow), the chromatosome stop that transiently blocks MNase digestion. This chromatosome stop was weaker for the nucleosomes containing the C-terminally truncated H2A, confirming the lower binding affinity of H1 towards the nucleosomes, but still maintaining the correct binding mode.

Altogether, these results point towards a novel role of the H2A C-terminus in mediating binding of the linker histone to the nucleosomes. Our results also suggest that the C-terminus of H2A can further regulate chromatin structure and dynamics via modulation of its interaction with the linker histone H1.

## Discussion

To unravel the functional role of the so far unstudied H2A C-terminal tail, we used H2A with partial or complete deletion of this tail for complementary *in vitro* and *in vivo* experiments. Cells expressing C-terminally truncated H2A were sensitive to stress, uncovering an important role of the tail in cellular homeostasis. By exploring the molecular interactions for which the H2A C-terminus is important, we identified three crucial functions: The H2A C-terminus (i) can affect local chromatin structure via its intrinsic modulation of nucleosome stability and positions, (ii) can regulate chromatin remodeling, as evident from the impairment of nucleosome repositioning by SNF2H and ISWI and (iii) can interact with the linker histone H1, which has implications for the mode of H1 binding to the nucleosome and presumably also higher order chromatin folding. Interestingly, many of these effects are, at least to some extend, already observed when truncating only the protruding part of the H2A tail. This demonstrates a crucial function not only of the DNA-passing region, but also of the protruding part of the tail. Additionally, the involvement of the H2A C-terminal tail in chromatin remodeling and as a protein interaction module indicates a role of the H2A tail beyond a mere structural function in stabilizing the nucleosomal core particle.

Histone octamers containing only H2A1-122 or H2A1-114 can assemble and reconstitute chromatin *in vitro*, demonstrating that the H2A C-terminal tail is not essential for chromatin formation (Figure 4). Mononucleosomes reconstituted on a linear DNA fragment revealed that positioning on the linear DNA and relative occupancy were different in nucleosomes containing H2A1-114 and also to a lesser extent with H2A1-122 (Figure 4A). Indeed, thermal shift assays further confirmed that the deletion of the C-terminal tail led to a drastic increase in the rate of nucleosome repositioning (Figure 4A, bottom panel). A recent study by Fink et al. [39] showed that in *S. cerevisae* the outermost part of the H2A C-terminal tail has no general effect on chromatin organisation. However, yeast H2A is most similar to mammalian H2AX, differing from canonical H2A in the last 11 amino acids of the C-terminal tail. Furthermore, in this study only the last four amino acids that are not present in mammalian canonical H2A were deleted.

Our findings suggest that the C-terminal tail of H2A can infer local changes in chromatin structure. This is supported by the analysis of its dynamic structure in the MD simulations: interactions of the H2A C-terminus and the DNA persisted during the ∼20 ns simulation time periods. These contacts occur next to the entry-exit sites of the linker DNA and might help to counteract unwrapping of the DNA. Interestingly our MD simulations show that these interactions are very transient and more dynamic than those that occur between the H3 N-terminus and the DNA. Therefore an additional mechanism might contribute to the stabilizing effect of the H2A C-terminus. Interestingly, the αN helix of H3 is situated closely to the C-terminal tail of H2A (Figure S2) [2]. Mutations in this αN helix have a negative effect on nucleosome stability and lead to an increase in thermal shifting and histone dimer exchange [25], suggesting that the instability of the interactions between the H3-H4 tetramer and the H2A C-terminal deleted-H2B dimers within the nucleosome can contribute to the destabilization of the nucleosomes.

### The H2A C-terminal tail is necessary for efficient chromatin remodeling

Among the remodeling machines tested here only ACF was effectively translocating H2A1-114 containing nucleosomes. ISWI and SNF2H failed to efficiently reposition nucleosomes lacking the complete H2A C-terminus (Figure 4B and 4C), despite these nucleosomes being less stable in our thermal shift assays. This unexpected reduced ATP-dependent chromatin remodeling of nucleosomes containing H2A1-114 may be explained by the position of the H2A C-terminus. The ISWI machines contact the nucleosomes at the linker DNA and within the first ∼50 bp of the nucleosomes [34]. The H2A C-terminus is close to this remodeler-nucleosome contact site and may thus influence the remodeler-nucleosome interaction and/or their affinity. The isolated ISWI and SNF2H ATPases were not sufficient to overcome the lack of the H2A C-terminus. However, the whole ACF complex did not discriminate between the nucleosomal substrates. The ACF complex contains two molecular motors that are linked by the Acf1 subunits and the remodeling activity of its ISWI subunit is largely enhanced [35], [37]. Additionally ACF binds with higher affinity to nucleosomal DNA. These features of ACF could help to overcome obstacles for remodeling. It is noteworthy that our assay does not distinguish between changes in remodeler-nucleosome affinities and reduced kinetics of the remodeling reaction due to the truncation of the H2A C-terminal tail.

Chromatin remodeling and decondensation are also important for efficient DNA repair [40] and stress response. The remodeling complexes allow the switching of local chromatin structures and the associated gene activity in response to external signals, as those machines determine nucleosome positions *in vitro* and *in vivo* [36], [41]–[43]. Therefore the inability of the nucleosomes containing the tail-less H2A to be recognized and remodeled, combined with the effects on nucleosome dynamics and H1 binding could—at least partially—explain the stress sensitivity of cells expressing truncated H2A.

It is important to note that we did not observe induction of cell death, apoptosis, or senescence (data not shown) in cells expressing C-terminally truncated H2A, arguing for specific effects on stress response rather than a generally reduced viability. In line with this it has recently been shown that a truncation of the H2A C-terminal tail by 8 amino acids can result in a specific change of gene expression profile [44], however the underlying mechanism remained unclear. This points towards a specific role of the H2A C-terminus in gene expression, stress-response and chromatin integrity that are mediated through the effects of the C-terminal tail on chromatin dynamics.

### The H2A C-terminus as a new targeting domain for H1?

In an unbiased screen we identified the linker histone H1 as a novel binding partner *in vivo*. In line with this our *in vitro* studies demonstrated decreased binding of H1 to mononucleosomes lacking the H2A C-terminus (Figure 5C). At higher molar ratios of H1 a chromatosome can be formed in the absence of the H2A C-terminal tail (Figure 5D), indicating that the H2A C-terminus mediates efficient H1 binding, but seems not to determine the binding mode.

This identification of the C-terminus of H2A as a new H1 targeting domain H1 demonstrates a novel function beyond the direct stabilization of nucleosomal core particles. In support of this a purification of the histone H1.2 found it in a complex with free H2A [45]. In order to evaluate what type of interactions between the two proteins would be possible in the context of the chromatosome we investigated the available model structures with respect to their potential for protein-protein interactions between the H2A C-terminus and H1 [11]–[15].

For a model based on the location of the globular H1 domain as proposed by Brown et al. [12] we found an interaction between H2A and the C-terminus of H1 that was stable in the molecular dynamics simulations if the C-terminal region of H2A is in a *trans* conformation, in which also interactions with the linker DNA are increased (Figure 1A, Figure S2). This illustrates how the H2A C-terminus can affect both the stability of the nucleosomal DNA and, at the same time, can determine its ability to interact with the linker histone and potentially also other chromosomal proteins. It has to be noted that the lack of high resolution structural information for the positioning of the H1 globular domain currently precludes a systematic investigation of these effects.

The role of linker histone H1 in chromatin compaction and higher order chromatin organization has been well recognized [46]. Its interaction with H1 establishes the C-terminal tail of H2A as an important player in chromatin dynamics *in vivo*. In addition, not only its importance for a stable nucleosome structure, but also for nucleosome remodeling points to a crucial role in targeting nucleosome positioning and regulating chromatin organization. Moreover, our results on the multiple functions of the H2A C-terminus predict that in H2A variants the differences in the C-terminal parts can fine-tune the function of H2A.

## Material and Methods

### Recombinant expression and purification of histones

Expression plasmids for human histones were a kind gift from J.D. Parvin. The H2A deletion constructs were cloned using the following oligonucleotides: TCGGATCCATGTCTGGGCGTGGCAAGC (forward), TACTCGAGTCACTT GCCCTTGGCCTTGTGG (reverse H2A1-129), TACTCGAGCTAACTCTCG GTCTTCTTAGGCAG (reverse H2A1-122), TACTCGAGCTACACGGCCTG GATGTTAGGAAGG (reverse H2A1-114). Recombinant expression and purification of histones from inclusion bodies was done as described previously [47]. After unfolding, 2 mg of each of the four core histones were mixed. Completely assembled octamers were separated from H3-H4 tetramers and H2A-H2B dimers by gel filtration over a Superose6 column (GE) equilibrated with refolding buffer.

### *In vitro* reconstitution of chromatin

Nucleosome reconstitution on linear DNA fragments was done as described [48]. DNA fragments generated by PCR were mixed with an appropriate amount of recombinant histone octamers and adjusted to a final salt concentration of 2 M NaCl. Typical assembly reactions contained 4 µg DNA, 250 ng plasmid DNA serving as competitor, histones and 200 ng µl^−1^ BSA in a total volume of 40 µl.

ATP-dependent nucleosome remodeling assays were performed according to standard procedures [34], [49]. Briefly, recombinant SNF2H, ISWI and ACF were expressed in Sf9 cells and prepared according to standard procedures. For the remodeling reactions, reconstituted nucleosomes were incubated for 90 minutes at 26°C with rising amounts of remodeler enzymes in Ex40 buffer (20 mM Tris HCl pH 7.6, 1.5 mM MgCl_2_, 0.5 mM EGTA, 10% (v/v) Glycerin, 40 mM KCl) containing 1 mM ATP. To stop the reactions, 1 µg of plasmid DNA was added and the reactions further incubated for another 5 minutes. Nucleosome positions were analyzed on a native 5% polyacrylamide gel (0.5× TBE) and visualized by ethidium-bromide staining.

### Thermal mobilization of nucleosomes

Mobility shift assays utilizing thermally induced movement of nucleosomes were carried out as described [50]. The DNA fragment for nucleosome assembly was generated by PCR using the AB_485 plasmid (kind gift from A. Flaus) and appropriate primers. For thermal shifts, 400 ng of mononucleosomal DNA were incubated in a total volume of 20 µl (50 mM Tris/HCl pH 7.6) at 45°C for 2, 4, 8, 16, 32, 64 and 128 minutes. Nucleosome positions were analyzed on a native 5% polyacrylamide gel (0.5× TBE) and visualized by ethidium-bromide staining. Quantification was done with the Multi Gauge Software (FUJIFILM).

### Stepwise salt elution of chromatin bound proteins

To assay for the salt stability of chromatin bound proteins, a stepwise salt elution was performed as described previously [17]. The extracts were analyzed by Western blot for the presence of GFP-H2A with an antibody specific for GFP or endogenous H2A (Upstate).

### Chromatin fractionation

Chromatin was fractionated as described previously [28], [29]. The nuclei were resuspended in 200 µl NB (20 mM Tris pH 7.6, 70 mM NaCl, 20 mM KCl, 5 mM MgCl_2_, 3 mM CaCl_2_) and incubated at 25°C for 10 min. 3000 U of micrococcal nuclease (Fermentas) were added and 60 µl samples were removed after digestion. The digestion was stopped by incubation on ice for 10 min. After centrifugation at 20000 g the supernatant S1 containing the accessible chromatin fraction was collected. The remaining pellet was resuspended in 200 µl 2 mM EDTA, incubated on ice for 10 min and centrifuged as before. The supernatant S2 contained the inaccessible chromatin fraction. The pellet fraction P was obtained by resuspending the last pellet in 2 mM EDTA.

### Cultivation of cells

All cells were cultivated at 37°C and 95% humidity with 5% CO_2_. *Dulbecco's Modified Eagle's Medium* (DMEM), high glucose (4,5 g/l) with 10% foetal calf serum (FCS, Perbio) and 1% L-Glutamine (200 mM) was used. Cells were transfected with plasmids pcDNA3-GFP (Invitrogen) containing H2A1-129, H2A1-122 or H2A1-114 and selected for stable expression with G418 (Calbiochem).

For cell cycle synchronization, cells were incubated with medium containing 2,2 mM thymidine for 13–15 h, released for 9 h and blocked again for 13–15 h. After releasing the cells from the block, samples were collected every 2 h for FACS analysis of the cell cycle stage distribution. To monitor the growth of asynchronous cells or after the cell cycle block every 24 h the cell number was determined in triplicates.

To determine the survival capability of cells, a colony forming assay was performed as described [51]. Cells were seeded at 500 cells per 10 cm dish and incubated for 10 days. The colonies were fixed with 3,7% formaldehyde and stained with 0,1% (w/v) crystal violet. The number of colonies was determined. For DNA damage experiments, the cells were treated with 1 µM camptothecin (CPT) for 30 min or 1 mM hydroxyurea (HU) for 4 h.

### Immunostaining and metaphase spreads

Immunostaining on fixed cells was performed as described [52] using GFP (molecular probes), H3K4me3 (Diagenode) and H3K9me3 (Upstate) antibodies. Chromosome spreads were prepared as described previously [53]. Cells were arrested in mitosis by incubation with 1 µg ml^−1^ Colcemid for 3 h prior to harvesting. 4*10^4^ cells were cytospun for 10 min at 1800 rpm and the slides directly put into KCM (120 mM KCl, 20 mM NaCl, 10 mM Tris pH 8.0, 0.5 mM EDTA, 0.1% Triton X-100) for 10 min at room temperature. The chromosomes were fixed by incubation in KCM +4% formaldehyde.

### Fluorescence recovery after photobleaching (FRAP) analysis

The experiments were performed on a Zeiss LSM 510 confocal microscope with a 100x/1.4 numerical aperture Plan Apochromat oil objective and 5x digital zoom. GFP was excited with the 488 nm line of an argon laser, and GFP emission was monitored above 505 nm as described previously [26]. For quantification, the total fluorescent intensities of a region of interest in the bleached area and in the total nuclear area were monitored using Zeiss software. Background fluorescence (BG) was measured in a random field outside the cells. The relative fluorescence intensity double normalized to the pre-bleach value was calculated at each time point as: *I*_rel_  =  (*T*_o_ - BG)×(*I*_(t)_-BG)/(*T*_(t)_ - BG)×(*I*_o_ - BG) where *T*_o_ is the average intensity of the entire nucleus during pre-bleach, and *I*_o_ is the average intensity of the region of interest during pre-bleach. For quantification 10–15 cells were used.

### Protein interaction assays

For purification of proteins interacting with the H2A C-terminal tail, HeLa cells stably expressing 8x repeats of the C-terminal 17 aa of H2A with HA-Flag tags were used. Purification was done from approx. 3*10^8^ cells as described [54]. After affinity purification with anti-Flag sepharose (Sigma), the bound proteins were eluted with Flag peptide and analyzed by mass spectrometry (Taplin Mass Spectrometry Facility, Harvard). For co-immunoprecipitation, the bound proteins were analyzed by immunoblotting with a H1 specific antibody (Abcam).

### Characterisation of H1-nucleosome interactions

The binding of H1 protein to chromatin was analyzed as described previously [10]. Human H1 expression constructs were a kind gift of D. Doenecke. Mononucleosomes were reconstituted on 208 bp DNA fragments containing the 601 positioning sequence [55]. 300 ng of nucleosomal DNA were incubated with increasing amounts of H1 in TE buffer containing 50 mM NaCl. The reactions were incubated at 23°C for 30 minutes and then analyzed on native 7.5% polyacrylamide gels (0.5× TBE). Nucleosome positions were visualized by ethidium bromide staining. Reconstituted mononucleosomes (with and without H1) were digested with Micrococcal Nuclease (MNase) for 1 to 8 minutes in the presence of 3 mM CaCl_2_. The reactions were stopped with 4 µl of stop buffer (4% SDS, 100 mM EDTA). Proteinase K and glycogen were added and deproteinization was carried out for 1 hour at 45°C. DNA was purified, analysed on 7.5% polyacrylamide gels and visualized by ethidium-bromide staining.

### Modelling H2A interactions

Molecular Dynamics simulations were performed in explicit water and 150 mM NaCl with the NAMD software [56] for a nucleosome particle with linker DNA extracted from the tetranucleosome structure [57]. Start structures for the MD simulations were generated by minimization with slowly released constraints on the structure atoms (150 ps), heating of the solvent to 300 K (50 ps), volume adjusting in NPT ensemble (100 ps) and a final 150 ps NVE simulation. Subsequently, MD simulations of two times 20 ns (nucleosome) and 2 ns (complex with H1) were conducted to determine the protein-protein and protein-DNA contacts of the H2A C-terminus.

### Nucleosome preparation by sucrose gradient

The nuclei of 1*10^7^ cells were prepared and digested with 10 u MNase per 50 µg DNA for 10 min at 14°C. The oligonucleosomes were separated on a 5–40% sucrose density gradient by centrifugation for 14 h at 36000 rpm. The gradient was fractionated into 20 fractions and the fractions were precipitated with methanol and chloroform according to Wessel-Flügge and the DNA and protein collected and analyzed.

## Supporting Information

Figure S1 Incorporation of GFP-H2A in chromatin. (A) GFP-H2A fusions are assembled into nucleosomes. Nuclei were prepared from the GFP-H2A1-129, GFP-H2A1-122 and GFP-H2A1-114 expressing cell lines and MNase digested. The chromatin from approx. 1×10^−7^ cells was loaded on a 5%–40% sucrose gradient with 0.6M NaCl. 20 fractions each were analysed. The presence of the GFP fusion in the octamers was detected with GFP antibodies and of DNA by ethidium bromide staining. Upper panels: immunoblots against GFP and H3. Lower panels: DNA samples stained with ethidium bromide. (B) GFP-H2A can be found in both euchromatin and heterochromatin compartments. Confocal analysis of HEK293 cells stained with H3K9me3 (as a marker for heterochromatin, upper panel) and H3K4me3 (as a marker for sites of active transcription, lower panels) (red). GFP is shown in green, DAPI staining in black and white on the left. Note that the GFP signal follows in general the DAPI signal and that there is no preferential enrichment of GFP-H2A in eu- or heterochromatin. Scale bar represents 10 µm.
(10.13 MB TIF)

Figure S2 Interactions of the H2A C-terminus with linker histone H1. Model of the interaction between H1 and the H2A C-terminus. Top: Histones H2A and H3 and DNA in the nucleosome are shown. DNA, transparent light grey; H3, dark grey; H1, yellow; H2A in blue. The binding site of the globular domain of H1 is based on a previously proposed model that has been extended with the C-terminal domain of H1 [12], [15]. This structure was then subjected to molecular dynamics simulations to evaluate interactions of the H2A C-tail with H3 and H1. Bottom: Enlarged view of the interactions between H2A and H1. H1 atoms within the C-terminal domain of the protein that, based on the molecular dynamics simulations, could interact with H2A C-terminus are shown with their van der Waals volumes. According to this model the H1 interactions are favoured for the trans conformation of the H2A-C tail (see Figure 1A for trans or cis conformation with respect to the βC part of the H2A). The trans conformation has increased contacts with the linker DNA. Thus, by shifting the trans-cis equilibrium via the H2A - H1 interaction to the trans conformation, the presence of H1 might also inhibit unwrapping of the DNA thereby increasing nucleosome stability.
(2.19 MB TIF)
