# Supplementary figures and images for: Histone H2A C-Terminus Regulates Chromatin Dynamics, Remodeling, and Histone H1 Binding
Source: PLoS Genet. 2010 Dec 9;6(12):e1001234. doi: 10.1371/journal.pgen.1001234 (PMC3000355; doi:10.1371/journal.pgen.1001234)

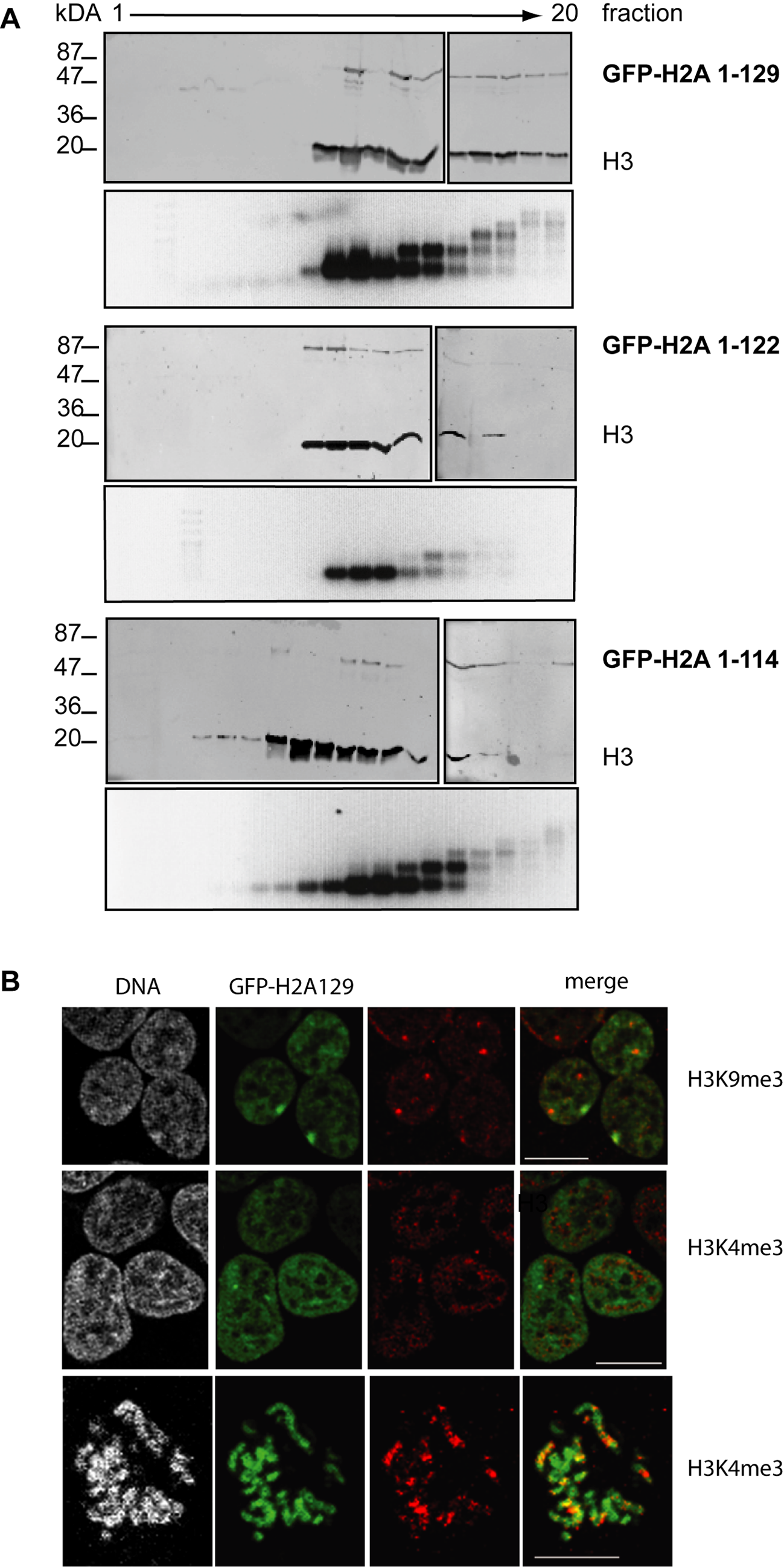

Supplement: Figure S1 — Incorporation of GFP-H2A in chromatin. (A) GFP-H2A fusions are assembled into nucleosomes. Nuclei were prepared from the GFP-H2A1-129, GFP-H2A1-122 and GFP-H2A1-114 expressing cell lines and MNase digested. The chromatin from approx. 1×10^−7^ cells was loaded on a 5%–40% sucrose gradient with 0.6M NaCl. 20 fractions each were analysed. The presence of the GFP fusion in the octamers was detected with GFP antibodies and of DNA by ethidium bromide staining. Upper panels: immunoblots against GFP and H3. Lower panels: DNA samples stained with ethidium bromide. (B) GFP-H2A can be found in both euchromatin and heterochromatin compartments. Confocal analysis of HEK293 cells stained with H3K9me3 (as a marker for heterochromatin, upper panel) and H3K4me3 (as a marker for sites of active transcription, lower panels) (red). GFP is shown in green, DAPI staining in black and white on the left. Note that the GFP signal follows in general the DAPI signal and that there is no preferential enrichment of GFP-H2A in eu- or heterochromatin. Scale bar represents 10 µm. (10.13 MB TIF) [file pgen.1001234.s001.tif]

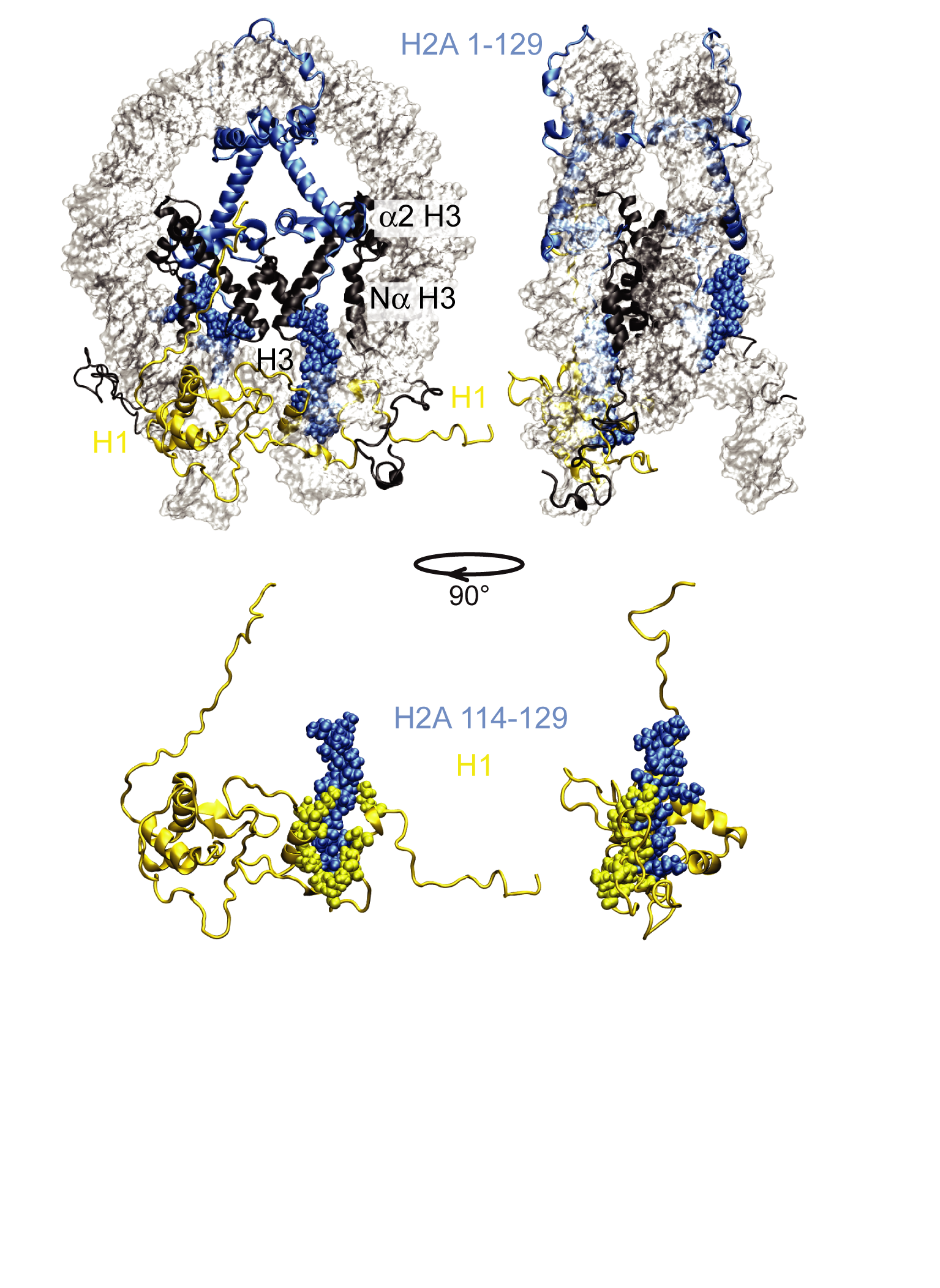

Supplement: Figure S2 — Interactions of the H2A C-terminus with linker histone H1. Model of the interaction between H1 and the H2A C-terminus. Top: Histones H2A and H3 and DNA in the nucleosome are shown. DNA, transparent light grey; H3, dark grey; H1, yellow; H2A in blue. The binding site of the globular domain of H1 is based on a previously proposed model that has been extended with the C-terminal domain of H1 [12], [15]. This structure was then subjected to molecular dynamics simulations to evaluate interactions of the H2A C-tail with H3 and H1. Bottom: Enlarged view of the interactions between H2A and H1. H1 atoms within the C-terminal domain of the protein that, based on the molecular dynamics simulations, could interact with H2A C-terminus are shown with their van der Waals volumes. According to this model the H1 interactions are favoured for the trans conformation of the H2A-C tail (see Figure 1A for trans or cis conformation with respect to the βC part of the H2A). The trans conformation has increased contacts with the linker DNA. Thus, by shifting the trans-cis equilibrium via the H2A - H1 interaction to the trans conformation, the presence of H1 might also inhibit unwrapping of the DNA thereby increasing nucleosome stability. (2.19 MB TIF) [file pgen.1001234.s002.tif]
